# Perinatal Risks Associated with Early Vanishing Twin Syndrome following Transfer of Cleavage- or Blastocyst-Stage Embryos

**DOI:** 10.1155/2016/1245210

**Published:** 2016-12-22

**Authors:** Nigel Pereira, Katherine P. Pryor, Allison C. Petrini, Jovana P. Lekovich, Jaclyn Stahl, Rony T. Elias, Steven D. Spandorfer

**Affiliations:** ^1^The Ronald O. Perelman and Claudia Cohen Center for Reproductive Medicine, Weill Cornell Medicine, New York, NY, USA; ^2^Department of Obstetrics and Gynecology, Weill Cornell Medical College, New York, NY, USA

## Abstract

*Objective*. To investigate whether the perinatal risks associated with early vanishing twin (VT) syndrome differ between cleavage- or blastocyst-stage embryo transfers (ET) in fresh in vitro fertilization (IVF) cycles.* Methods*. Retrospective, single-center, cohort study of IVF cycles with fresh cleavage- or blastocyst-stage ETs resulting in a live singleton birth. The incidence of preterm birth (PTB), low birth weight (LBW), and very low birth weight (VLBW) was compared between cleavage- and blastocyst-stage ET cycles complicated by early VT.* Results*. 7241 patients had live singleton births. Early VT was observed in 709/6134 (11.6%) and 70/1107 (6.32%) patients undergoing cleavage-stage and blastocyst-stage ETs, respectively. Patients in the blastocyst-stage group were younger compared to the cleavage-stage group. The cleavage-stage group had a similar birth weight compared to the blastocyst-stage group. There was no difference in the incidence of PTB (9.87% versus 8.57%), LBW (11.1% versus 11.4%), or VLBW (1.13 versus 1.43%) when comparing the cleavage-stage early VT and blastocyst-stage early VT groups, even after adjustment with logistic regression.* Conclusions*. Our study highlights that the adverse perinatal risks of PTB, LBW, and VLBW associated with early VT syndrome are similar in patients undergoing cleavage-stage or blastocyst-stage ETs during fresh IVF cycles.

## 1. Introduction

The use of in vitro fertilization (IVF) to overcome infertility continues to increase globally. Over 4,000,000 IVF cycles were initiated worldwide between 2008 and 2010, resulting in the birth of 1,144,858 children [1]. A majority of these IVF cycles involve the transfer of >1 embryo to achieve a pregnancy, often contributing to the pathogenesis of multiple pregnancies, which occurs in up to 23.1% of fresh IVF cycles [1]. With successful advances in culture media, there has been a practice shift in IVF from cleavage-stage to blastocyst-stage embryo transfers (ETs) [2, 3]. Thus, the extended culture of embryos to the blastocyst-stage allows for the selection of fewer embryos for ET compared to cleavage-stage ETs [2, 3]. Yet, the number of single ET cycles remains relatively low (i.e., 21.4% in 2013 (United States) and 30.0% in 2010 (globally) [1, 4]), suggesting that transfer of >1 embryo, either cleavage-stage or blastocyst-stage, remains the norm in IVF cycles.

Approximately one out of every 10 singleton IVF pregnancies are thought to originate from a twin gestation [5] in a phenomenon known as vanishing twin (VT) syndrome [6]. Existing data suggest higher perinatal risks such as low birth weight and preterm birth in the surviving twin of VT syndrome [7–13]. However, it is important to note that these data are primarily generated from studies involving the transfer of >1 cleavage-stage embryo to achieve a pregnancy. Although one study has previously reported a decreased risk of VT syndrome in blastocyst-stage ET when compared to cleavage-stage ET [14], it is currently unknown whether early VT syndrome is associated with different perinatal risks in blastocyst or cleavage-stage ET cycles. Thus, the primary objective of the current study is to investigate whether the perinatal risks associated with early VT syndrome differ between cleavage- and blastocyst-stage ETs in fresh IVF cycles.

## 2. Materials and Methods

### 2.1. Cycle Inclusion Criteria

All fresh IVF cycles with transfer of cleavage- or blastocyst-stage embryos between 2004 and 2013 at the Ronald O. Perelman and Claudia Cohen Center for Reproductive Medicine were analyzed for potential inclusion. Only patients with live singleton birth were included. The diagnosis of early VT was made based on criteria described by Petrini et al. [15]. In brief, patients with a positive *β*-human chorionic gonadotropin (hCG) level on cycle day (CD) 28 underwent a transvaginal sonogram on CD 49 to record the number of fetal poles with cardiac activity in the respective gestational sacs [15]. Patients with spontaneous in utero reduction of one or more embryos before CD 49 were considered to have an early VT. For this study, we only included embryos that spontaneously reduced within the first trimester. All patients utilizing donor oocytes or undergoing frozen-thawed ET were excluded. The retrospective cohort study protocol was approved by the Weill Cornell Medical College Institutional Review Board.

### 2.2. Clinical and Laboratory Protocols

Previously described protocols for ovarian stimulation, ovulatory trigger, and oocyte retrieval were utilized [16]. Ovarian stimulation began on CD 2 of menses with gonadotropins (Follistim, Merck, Kenilworth, NJ, USA, or Gonal-F, EMD-Serono Inc., Rockland, MA, USA, and Menopur, Ferring Pharmaceuticals Inc., Parsippany, NJ, USA). Dosing of gonadotropins was based on patient age, body mass index (BMI, kg/m^2^), antral follicle count, and previous response to stimulation, if any. Ovulation was suppressed with daily injection of 0.25 mg Ganirelix Acetate (Merck, Kenilworth, NJ, USA). The hCG trigger (Pregnyl, Merck, Kenilworth, NJ, USA) was given when the two lead follicles attained a mean diameter >17 mm. Oocyte retrieval was performed under conscious sedation using transvaginal sonogram guidance approximately 34-35 hours after the hCG trigger. The retrieved oocytes were fertilized using intracytoplasmic sperm injection (ICSI) or conventional in vitro insemination depending on the male partner's semen analysis [17]. All embryos were cultured in in-house culture media [18]. Cleavage-stage embryos were graded based on the Veeck criteria, while the blastocyst-stage embryos were graded based on criteria described by Gardner and Schoolcraft [19]. The majority of patients underwent cleavage-stage ET, while those with several good-quality cleavage-stage embryos on day 3 were eligible for blastocyst-stage ET on day 5 [20]. All ETs were performed with Wallace catheters (Smiths Medical Inc., Norwell, MA, USA).

### 2.3. Study Variables

Baseline demographics recorded for all patients included age, parity, BMI (kg/m^2^), and infertility diagnosis. Ovarian stimulation parameters recorded were total days of ovarian stimulation, total dosage of gonadotropins administered (IU), peak estradiol (E_2_) level (pg/mL), peak endometrial thickness (mm), and total number of oocytes retrieved. The mean number of embryos transferred per patient was recorded, based on which implantation rates were calculated (i.e., the number of embryos with cardiac activity detected via transvaginal sonography on CD49 out of the total number of embryos transferred). Perinatal outcomes recorded for all live births were mode of delivery, preterm birth (PTB), low birth weight (LBW), very low birth weight (VLBW), and term LBW. Any live birth at <37 weeks of gestational age was defined as PTB [21]. PTB between ≥34 and <37 weeks of gestation was defined as late PTB, while that occurring at <34 weeks of gestation was classified as early PTB [21]. Birth weights <2,500 g and <1,500 g irrespective of gestational age were classified as LBW and VLBW, respectively [22]. Any live singleton born ≥37 weeks of gestational age, but with a birth weight of <2,500 grams, was considered a term LBW singleton [21, 22].

### 2.4. Statistical Analyses

Continuous variables were checked for normality using Shapiro-Wilk's test and expressed as mean ± standard deviation (SD). Categorical variables were expressed as number of cases (*n*) and percentage (%). Nonparametric variables were expressed as median (interquartile range [IQR]). Independent* t*-tests, Wilcoxon rank-sum tests, and Chi-square tests were used as indicated. Odds ratios (OR) with 95% confidence intervals (CI) were calculated for the incidence of PTB, LBW, VLBW, and term LBW and were adjusted with multiple logistic regression, controlling for the following variables: age (<35 years versus ≥35 years); total days of ovarian stimulation (<9 days versus ≥9 days); total gonadotropins administered (<2000 IU versus ≥2000 IU); peak E_2_ level (<2000 versus ≥2000 pg/mL); total number of oocytes (<10 versus ≥10); and blastocyst-stage ET (yes versus no). A* P *value < 0.05 was considered statistically significant. All statistical analyses were performed using STATA version 13 (College Station, TX: StataCorp LP).

## 3. Results

A total of 7241 patients had live singleton births during the study period. Cleavage- and blastocyst-stage ETs contributed to live singleton births in 6134 (84.7%) and 1107 (15.3%) patients, respectively. Early VT was observed in 709 (11.6%) and 70 (6.32%) patients undergoing cleavage-stage and blastocyst-stage ETs, respectively, which represented a 0.51 times lower odds of early VT in the blastocyst-stage group compared to the cleavage-stage group (OR 0.51; 95% CI 0.19–1.41). Of note, these odds are similar to the odds reported by Fernando et al. [14].

Table 1 compares the baseline demographics of all patients undergoing cleavage-stage or blastocyst-stage ETs with early VT. While there were no differences in the mean parity, BMI, and distribution of infertility diagnoses, patients in the blastocyst-stage group (36.2 ± 1.01 years) were younger compared to the cleavage-stage group (38.1 ± 2.98 years; *P* < 0.001).

As highlighted in Table 2, patients undergoing blastocyst-stage ET required a lower dosage of gonadotropins and had higher peak E_2_ levels compared to the cleavage-stage group. Also, the yield of total and mature oocytes was higher in the latter group compared to the former. These differences suggested a more robust ovarian response in blastocyst-stage ET group. There were no differences in the total days of ovarian stimulation or peak endometrial thickness.

Table 3 summarizes the perinatal outcomes of patients undergoing cleavage-stage or blastocyst-stage ETs with early VT. The mean number of embryos transferred was higher in the cleavage-stage group (2.64 ± 0.91) compared to the blastocyst-stage group (1.69 ± 0.42). No difference in the mode of delivery, rate of term birth, rate of late PTB, or rate of early PTB was noted. The cleavage-stage group had a similar birth weight compared to the blastocyst-stage group (3187.1 ± 409.9 grams versus 3198.2 ± 387.2 grams; *P* = 0.82). Furthermore, the odds of LBW, VLBW, or term LBW were nonsignificant when comparing the groups.

Tables 4, 5, 6, and 7 highlight the adjusted OR for PTB, LBW, VLBW, and term LBW, respectively, after accounting for age, total gonadotropins administered, total days of ovarian stimulation, peak E_2_ level, total number of oocytes, and blastocyst-stage ET using multiple logistic regression. As evident from the aforementioned tables, no differences in the odds of adverse perinatal outcomes between the cleavage-stage and blastocyst-stage groups were noted.

## 4. Discussion 

Since the recognition of VT syndrome in the 1970s [23], several investigators have reported adverse perinatal outcomes such as LBW, PTB, low APGAR scores, and fetal malformations associated with VT syndrome in a myriad of clinical settings and modes of conception. Several investigators have postulated reasons for the association of perinatal risks and VT syndrome. La Sala et al. [6] proposed that VT syndrome is a subtype of single fetal demise in twins, resulting in blood shunting from the placenta of the surviving twin, ultimately leading to deleterious effects. Thus, increasing gestational ages of VT would be associated with heightened perinatal risks. For example, the rates of PTB (16.7%) reported by La Sala et al. [6] for VT syndrome occurring after 8 weeks of gestation are higher than the rates of PTB (9.9% for cleavage-stage ET and 8.6% for blastocyst-stage ET) reported in our study after early VT. Another theory suggests that chronic inflammation following VT reduction directly impacts the growth and progression of the surviving twin [10–12]. Finally, Mansour et al. [7] reported that adverse perinatal outcomes associated with VT syndrome may be mediated through early embryonic modification rather than a uterine or placentation etiology.

The impact of ET stage on perinatal outcomes in IVF cycles associated with early VT syndrome is currently unknown. Thus, our study evaluates the perinatal risks associated with early VT in two different implantation models—cleavage-stage ET and blastocyst-stage ET. Historically, cleavage-stage ET was considered standard in IVF-ET cycles, primarily due to limitations in our knowledge of stage-specific culture medium requirements and the low survival of embryos cultured past this stage [2, 3]. Advances in laboratory protocols have enabled extended culture with subsequent increase in blastocyst-stage ET. Yet, the ideal ET stage depends on several factors including consideration of short-term outcomes (including implantation and clinical pregnancy rates, cycle cancellation rates, and likelihood of survival of embryos to blastocyst-stage) and long-term outcomes (including live birth rates, multiple gestations, and perinatal outcomes). Despite improvements in extended culture and growing evidence to support greater use of blastocyst-stage ET, cleavage-stage ET is still utilized in the majority of IVF-ET cycles [2, 3].

Our study demonstrates that the incidence of early VT is lower in blastocyst-stage ET cycles compared to cleavage-stage ET cycles. However, the adverse perinatal risks of PTB, LBW, and VLBW associated with early VT syndrome are similar in patients undergoing cleavage-stage or blastocyst-stage ETs during fresh IVF cycles. Salient strengths of the current study include its large sample size and utilization of multiple logistic regression to account for several confounding variables. Despite these strengths, our study is not without limitations. First, though we attribute the perinatal risks seen in our population to early dizygotic twinning, the chorionicity of the pregnancies included in our study cohort was not evaluated. It is possible that monochorionic gestations, at least in part, may have contributed to the pathogenesis of the aforementioned perinatal risks. Second, given the retrospective nature of this study, we remain uncertain whether our findings would hold true in larger prospective settings.

While previous studies suggest that early VT syndrome confers increased perinatal risks in fresh IVF-ET cycles, out study emphasizes that these outcomes do not differ between cleavage- and blastocyst-stage ETs. It is important to note that the increased odds of LBW noted in our study are still significantly lower than the incidence of LBW associated with VT syndrome reported in previous publications [7–13]. While the stage of ET does not impact the perinatal risks associated with early VT, our study does highlight the need to perform single embryo transfers when possible to minimize adverse outcomes associated with the transfer of >1 embryo in IVF-ET cycles. Finally, patients with early VT syndrome should be counseled about potential perinatal risks. Ultrasonographic surveillance of the surviving twin to confirm adequate growth may be considered as a reasonable clinical strategy in such patients.

## Figures and Tables

**Table 1:** Baseline demographics of patients undergoing cleavage-stage ETs (*n* = 709) or blastocyst-stage ETs (*n* = 70) with early VT.

Parameter	Cleavage-stage (*n* = 709)	Blastocyst-stage (*n* = 70)	*P*
Age (years)	38.1 (±2.98)	36.2 (±1.01)	<0.001
Parity	0.71 (±0.21)	0.59 (±0.38)	0.11
BMI (kg/m^2^)	23.1 (±2.97)	22.9 (±1.83)	0.55
Infertility diagnoses			0.28
Ovulatory	198 (27.9%)	15 (21.4%)	
Tubal	38 (5.36%)	8 (11.4%)	
Endometriosis	37 (5.22%)	7 (10%)	
Male factor	262 (37.0%)	22 (31.4%)	
Idiopathic	39 (5.50%)	9 (12.9%)	
Other	135 (19.0%)	9 (12.9%)	

Data are presented as mean ± standard deviation, median (interquartile range), and *n* (%); BMI: body mass index.

**Table 2:** Comparison of ovarian stimulation parameters of patients undergoing cleavage-stage ETs (*n* = 709) or blastocyst-stage ETs (*n* = 70) with early VT.

Parameter	Cleavage-stage (*n* = 709)	Blastocyst-stage (*n* = 70)	*P*
Total days of ovarian stimulation	9.77 (±1.49)	9.41 (±1.12)	0.06
Total dosage of gonadotropins (IU)	2992.7 (±329.1)	2527.2 (±278.3)	<0.001
Peak E_2_ level (pg/mL)	1609.2 (±471.9)	2259.0 (±512.3)	<0.001
Peak endometrial thickness (mm)	10.8 (±2.12)	10.7 (±1.92)	0.71
Total number of oocytes	12 (9–15)	14 (9–17)	<0.001
Total number of mature oocytes	10 (7–13)	12 (7–15)	<0.001

Data are presented as mean ± standard deviation, median (interquartile range), and *n* (%); E_2_: estradiol.

**Table 3:** Comparison of perinatal outcomes in patients undergoing cleavage-stage ETs (*n* = 709) or blastocyst-stage ETs (*n* = 70) with early VT.

Parameter	Cleavage-stage (*n* = 709)	Blastocyst-stage (*n* = 70)	OR (95% CI)	*P*
Age (years)	38.1 (±2.98)	36.2 (±1.01)	—	<0.001
Number of embryos transferred	2.64 (±0.91)	1.69 (±0.42)	—	<0.001
Implantation rate	39.7%	43.2%	0.83 (0.47–1.46)	0.52
Mode of delivery				0.87
Vaginal	378 (53.3%)	38 (54.3%)	0.96 (0.59–1.57)	
Cesarean	331 (46.7%)	32 (45.7%)		
Term birth	639 (90.1%)	64 (91.4%)	0.86 (0.33–2.23)	0.75
Preterm birth				0.70
Late preterm	41 (5.78%)	4 (5.71%)	0.71 (0.12–4.12)	
Early preterm	29 (4.09%)	2 (2.86%)		
Overall birth weight (g)	3187.1 (±409.9)	3198.2 (±387.2)	—	0.82
LBW	79 (11.1%)	8 (11.4%)	0.97 (0.40–2.33)	0.95
VLBW	8 (1.13%)	1 (1.43%)	0.79 (0.07–9.43)	0.85
Term LBW	30 (4.69%)	4 (6.25%)	0.74 (0.22–2.53)	0.63

Data are presented as mean ± standard deviation, median (interquartile range), and *n* (%); LBW: low birth weight; VLBW: very low birth weight; OR: odds ratio; CI: confidence interval.

**Table 4:** Preterm birth and multiple logistic regression to account for confounding variables.

PTB	Standard error	Adjusted OR (95% CI)	*P*
Age (<35 versus ≥35 years)	0.21	0.87 (0.54–1.41)	0.58
Duration of ovarian stimulation (<9 versus ≥9 days)	0.36	0.83 (0.35–1.97)	0.67
Gonadotropin dose (<2000 versus ≥2000 IU)	0.86	0.93 (0.15–5.71)	0.94
Peak E_2_ level (<2000 versus ≥2000 pg/Ml)	0.24	1.12 (0.74–1.71)	0.57
Total number of oocytes (<10 versus ≥10)	0.13	0.70 (0.48–1.11)	0.16
Blastocyst-stage ET (yes versus no)	0.21	0.74 (0.42–1.28)	0.28

PTB: preterm birth; E_2_: estradiol; ET: embryo transfer.

**Table 5:** Low birth weight and multiple logistic regression to account for confounding variables.

LBW	Standard error	Adjusted OR (95% CI)	*P*
Age (<35 versus ≥35 years)	0.59	1.16 (0.43–3.14)	0.77
Duration of ovarian stimulation (<9 versus ≥9 days)	1.25	1.24 (0.17–8.96)	0.83
Gonadotropin dose (<2000 versus ≥2000 IU)	0.76	0.81 (0.13–5.04)	0.82
Peak E_2_ level (<2000 versus ≥2000 pg/Ml)	0.30	0.86 (0.44–1.69)	0.66
Total number of oocytes (<10 versus ≥10)	0.20	0.93 (0.18–4.13)	0.77
Blastocyst-stage ET (yes versus no)	0.11	0.85 (0.66–1.99)	0.20

LBW: low birth weight; E_2_: estradiol; ET: embryo transfer.

**Table 6:** Very low birth weight and multiple logistic regression to account for confounding variables.

VLBW	Standard error	Adjusted OR (95% CI)	*P*
Age (<35 versus ≥35 years)	0.47	1.04 (0.17–6.30)	0.97
Duration of ovarian stimulation (<9 versus ≥9 days)	0.54	0.96 (0.19–5.44)	0.75
Gonadotropin dose (<2000 versus ≥2000 IU)	0.41	0.71 (0.44–6.56)	0.44
Peak E_2_ level (<2000 versus ≥2000 pg/Ml)	0.19	0.81 (0.55–1.19)	0.29
Total number of oocytes (<10 versus ≥10)	0.26	0.98 (0.59–1.63)	0.94
Blastocyst-stage ET (yes versus no)	0.40	0.88 (0.36–2.17)	0.78

VLBW: very low birth weight; E_2_: estradiol; ET: embryo transfer.

**Table 7:** Term low birth weight and multiple logistic regression to account for confounding variables.

Term LBW	Standard error	Adjusted OR (95% CI)	*P*
Age (<35 versus ≥35 years)	0.46	0.86 (0.59–7.80)	0.76
Duration of ovarian stimulation (<9 versus ≥9 days)	0.73	0.92 (0.35–3.08)	0.33
Gonadotropin dose (<2000 versus ≥2000 IU)	0.42	0.83 (0.44–6.09)	0.38
Peak E_2_ level (<2000 versus ≥2000 pg/Ml)	0.32	0.92 (0.16–1.86)	0.48
Total number of oocytes (<10 versus ≥10)	0.39	0.94 (0.09–1.82)	0.25
Blastocyst-stage ET (yes versus no)	0.47	0.91 (0.28–3.83)	0.84

LBW: low birth weight; E_2_: estradiol; ET: embryo transfer.
